# Children with Usher syndrome: mental and behavioral disorders

**DOI:** 10.1186/1744-9081-8-16

**Published:** 2012-03-27

**Authors:** Jesper Dammeyer

**Affiliations:** 1 Department of Psychology, University of Copenhagen, Øster Farimagsgade 2A, 1353 København K, Denmark; 2 Centre for Deafblindness and Hearing Impairment, Aalborg, Denmark

**Keywords:** Deafblindness, Dual sensory loss, Mental and behavioral disorders, Usher syndrome, Psychiatry

## Abstract

**Background:**

Mental and behavioral disorders among adults with Usher syndrome have been discussed and reported in some case studies but no research has been reported on children with Usher syndrome.

**Methods:**

This article investigates the prevalence and characteristics of mental and behavioral disorders among 26 children, 3-17 years of age, with Usher syndrome.

**Results:**

Six of the 26 children were diagnosed with a mental or behavioral disorder (1 with schizophrenia and mild mental retardation, 1 with atypical autism and severe mental retardation, 1 with atypical autism and mild mental retardation, 1 with mild mental retardation, and 2 with conduct disorder). Another 3 children had had a mental or behavioral disorder previously in their childhood.

**Conclusion:**

Even though vision impairment first manifests in late childhood, some children with Usher syndrome seem to develop mental and behavioral disorders during childhood. The aetiology and treatment of mental and behavioral disorders among children with Usher syndrome are discussed. Children with Usher syndrome and their parents may need clinical support during early childhood to prevent development of mental and behavioral disorders.

## Background

Usher syndrome is characterized by deafness and a gradual loss of vision. The hearing loss is sensorineural, whereas the vision loss is associated with retinitis pigmentosa (RP), a degeneration of the retinal cells. Three subtypes of Usher syndrome have been found [1]. People with Usher I are congenitally deaf, and start to lose vision early in life. They also face balance difficulties due to vestibular system problems. Individuals with Usher II also experience hearing loss but are not profoundly deaf. They have no noticeable problems with their balance. Individuals with Usher syndrome III are not congenitally deaf, but gradually lose their sense of hearing and vision. Some individuals with Usher III experience balance difficulties while others do not [2,3]. Because of the balance difficulties many children with Usher take longer to learn to walk. Several genes have been associated with Usher syndrome. To date, seven genetic loci for Usher I (*USH1B-H*) have been mapped on chromosomes 14q32, 11q13.5, 11p15.1, 10q22.1, 21q21 and 10p-22, 17q24-25. Usher II is associated with mutations in three genes (USH2A, C-D). Mutations in the *USH2A *gene on chromosome 1q41 are the most common (85% of all cases with Usher II). Only one gene has been linked to Usher III. Usher III is caused by mutations in the USH3A (clarin-1) gene, mapped on 3q21-q25 [2].

A large amount of gene research has been conducted on Usher Syndrome [2], but only a small amount of research has focused on psychiatric and psychological issues. Different mental and behavioral disorders have been reported among adults. Psychosis (schizophrenic type picture) has been discussed in some case studies [4-12] and Hallgren [13] reported that 23% of 114 individuals with Usher syndrome were psychotic. However, Grøndahl and Mjøen [14] reported only one case of psychosis among 28 individuals with Usher syndrome and Nuutila [15] reported mere 4.5% of individuals with Usher syndrome to be psychotic in a study of 133 individuals. Mental retardation [16], anorexia nervosa [17] and ADHD [4] have also been discussed or reported upon in case-studies investigating Usher syndrome. Stress, anxiety, social isolation and depression are other mental health related issues that are proposed to be associated with Usher syndrome and the loss of hearing and vision [18,19]. Although a number of studies indicate that Usher syndrome might be associated with different mental and behavioral disorders, most of the studies are only based on case studies. Very few survey studies have been made and none within the last two decades.

In Scandinavia some clinical professionals have observed that children with Usher Syndrome face different kinds of mental and behavioral difficulties from early childhood. Until now no systematic studies have been reported on the prevalence of mental and behavioral disorders among children with Usher syndrome. The aim of this study is to investigate the prevalence and characteristics of mental and behavioral disorders among all children known to be suffering from Usher syndrome in Denmark.

## Methods

### Participants

All children and adolescents, 0-17 years of age, known to have Usher syndrome through the national service system in Denmark (Center for Deafblindness and Hearing Impairment in the city of Aalborg offer services for children with Usher syndrome in Denmark) were included in the study. This amounted to 26 children with Usher syndrome. All Usher syndrome diagnoses were clinically confirmed; RP by electroretinography (ERG) and hearing loss by standard hearing tests completed by a clinical audiologist. In 12 of the cases Usher syndrome was also confirmed by gene tests. Given an estimated prevalence of Usher syndrome at 3.5 to 6.2 per 100,000 inhabitants [20-22] the 26 children represent 36% to 65% of the total population of children with Usher syndrome in Denmark.

The children were between 3 and 17 years of age (*M *= 11, SD = 4.2) and 12 were boys. Twenty cases were diagnosed with Usher type I, and 4 cases with type II. There were no cases with type III. No real conclusions could be made for the remaining 2 cases (Usher type III was excluded because of stationary congenital hearing loss).

### Method and procedure

Information was obtained from the medical case records using a questionnaire form completed by deafblind consultants from the national service (Center for Deafblindness and Hearing Impairment) in collaboration with the parents. The consultants had close contact to the children and their families. Informed consent was obtained from parents or legal guardians. All agreed to participate.

Included in this study is information about the children regarding age, gender, language modality (oral language, sign-language, or preverbal communication), type of school/institution (school for deaf, blind or deafblind, special school not specialized for sensory loss, or mainstream school), mental and behavioral diagnoses as well as other medical diagnoses. Information about the mental and behavioral disorders was obtained from the case records. To ensure data validity, the case records were analyzed by the author to confirm that all diagnoses were made by medical specialists (psychiatrists) with knowledge of assessing children with dual sensory loss. The case records were also analyzed for whether standardized assessment procedures and tools had been used (Leiter-R [23] or a similar instrument for assessing mental retardation, Autism Diagnostic Observation Schedule-Generic (ADOS-G) [24] or a similar instrument for assessing autism spectrum disorders, etc.).

The Strengths and Difficulties Questionnaire (SDQ) [25] was also included. The SDQ is a short and easy behavioral screening questionnaire that asks about children's and teenagers' psychosocial difficulties. SDQ has been translated into Danish [26]. The version for teachers and parents was used. The SDQ contains items like "has at least one good friend," "often loses temper," and "often lies or cheats". The teacher or the parent is able to answer: "not true," "somewhat true," or "certainly true." The SDQ is designed for children from 4 to 16 years. The SDQ gives an overall score from 0 to 40, where 0 indicates no problems and 40 indicates a significant number of problems. Hintermair [27] has evaluated the psychometric properties of the SDQ and found it to be reliable and valid to use among children who are deaf or hard-of-hearing.

## Results

### About the population

Seventeen of the children went to a school for the deaf or hearing impaired, 5 to mainstream schools (including 3 of the 4 children with Usher type II), and 4 to special schools without a specialization for children with sensory impairment (2 children at schools for children with autism, 1 at a school for children with mental retardation, and 1 at a school for children with speech- and language disorder). Four children used sign language, 20 oral language, and 2 preverbal communication. The children using preverbal communication were the two children attending schools for children with autism.

One child was known to have Kartagener Syndrome and one Polycystic Ovary Syndrome.

### Mental and behavioral disorders and psychosocial difficulties

As listed in Table 1 six (23%) of the children with Usher Syndrome had a mental and behavioral disorder: 1 with schizophrenia and mild mental retardation (girl 16 years of age Usher type I), 1 with atypical autism and severe mental retardation (boy 15 years of age Usher type I), 1 with atypical autism and mild mental retardation (boy 11 years of age Usher type I), 1 with mild mental retardation (boy 12 years of age Usher type I), and 2 with conduct disorder (girl 14 years of age Usher type II, boy 13 years of age Usher type I). Among the remaining 20 children, 3 (12%) had previous a mental or behavioral disorder: 1 previous atypical autism (boy Usher type I), 1 previous eating disorder (boy Usher type I), and 1 previous conduct disorder (boy Usher type unknown). Seventeen children (65%) had never had any mental or behavioral disorders.

**Table 1:** Nine cases with Usher syndrome and current or previous diagnosed mental or behavior disorders

Age	Gender	Usher type	Mental or behavioral diagnose (age at diagnose)
16	Girl	I (type unknown)	Schizophrenia, mild mental retardation (8-)
15	Boy	I (type unknown)	Atypical autism, severe mental retardation (2-)
11	Boy	I (type unknown)	Atypical autism, mild mental retardation (3-)
12	Boy	I (type unknown)	Mild mental retardation (4-)
14	Girl	IIA	Conduct disorder (5-)
13	Boy	I (type unknown)	Conduct disorder (10-)
15	Boy	IB	Atypical autism (3-6)
16	Boy	I (type unknown)	Eating disorder (10-11)
11	Boy	(type unknown)	Conduct disorder (4-8)

Another 17 children had never had any mental or behavioral disorders

The SDQ was completed for each of the 20 children with-out any current mental or behavioral disorder except for two children (1 was younger than 4 years of age, 1 older than 16 years of age). Since the SDQ reflects symptoms of mental and behavioral disorders [25] it was not administered to the children with current mental or behavioral disorders. The mean score of the SDQ was 11.4 (*SD *= 5.7) with the lowest score of 2 and the highest of 25. In a normal Swedish population of 900 children, Smedje et al. [28] selected a cut-off score of 14 (90^th ^percentile) on the SDQ for both boys and girls. Using the cut-off score of 14 in this study shows that 5 children (27%) experienced psychosocial difficulties.

## Discussion

Mental and behavioral disorders were found among a quarter (23%) of the children with Usher syndrome. The types of disorders observed were not uniform and included atypical autism, mental retardation, schizophrenia, and conduct disorder. The number of individuals found with schizophrenia in this study (1 child) is congruent with results reported by Nuutila [15] and Grøndahl and Mjøen [14] using samples of 133 and 89 adults with Usher syndrome, respectively. Among the children without mental and behavioral disorders around one third was found to have psychosocial difficulties. All together, 11 children (42%) faced neither mental and behavioral disorder nor psychosocial difficulties.

Two major explanations for a higher incidence of mental and behavioral difficulties among children and adults with Usher syndrome can be stated. 1) Some argue that the progressive loss of vision results in severe stress and symptoms of mental and behavioral disorder. Case-studies report that mental and behavioral symptoms in patients with Usher syndrome developed simultaneously to the loss of vision. It has been suggested that they are reactive symptoms to severe stress [5,12,13]. In addition to stress-related responses imposed by progressive loss of vision, dual sensory loss may be associated with a higher prevalence of mental and behavioral disorders because of severe communicative difficulties [29]. Among adults with acquired deafblindness McDonnall [30] found that communication and social support was important in order to avoid the experience of depression. The communicative difficulties caused by the dual sensory loss, increase the risk of depression. Intervention by means of communication and language rehabilitation is found to be important in preventing mental and behavioral disorders and psychosocial difficulties. This rehabilitation is also important among children with hearing impairment alone [31-36] and people with deafblindness [29,37,38].

2) The second possible explanation for an association between Usher syndrome and mental and behavioral disorders is that some genes are predisposed to both Usher syndrome and for example schizophrenia [8]. Studies of families with more than one member with Usher syndrome and schizophrenia support this hypothesis [11,16,39]. But until now, no specific genes or mutations have been reported as being suspected candidates. Mental and behavioral disorders may also be a consequence of brain abnormalities associated with Usher syndrome [8]. Schaefer and colleagues [40] found that the measured volume of the brain was significantly smaller compared to normal controls and Koizumi et al. [10] found a global degeneration of the brain in one case with Usher syndrome and schizophrenia. Similar to the surveys by Nuutila [15] and Grøndahl and Mjøen [14] this study found that only some individuals with Usher syndrome had mental and behavioral disorders and the nomenclature of disorders was not uniform. Either different Usher genes are associated with different kinds of mental and behavioral disorders, or another explanation may be the necessary.

A developmental psychopathology perspective [41] of the dynamic interplay of physiological, genetic, social, cognitive, and cultural influences across time may be useful to understand the heterogenic picture of mental and behavioral disorders among children with Usher syndrome. Given the combination and number of disabilities (hearing, vision and balance), children with Usher syndrome may face more barriers to language, social and cognitive development compared to children with hearing loss alone. Even minor vision impairment may in combination with congenital hearing loss increase the child's difficulties developing useful language abilities. Due to the sum of barriers, children with Usher syndrome may become more vulnerable towards developing mental and behavioral disorders. More research is needed to learn more about this dynamic interplay.

Several authors have asserted that there is an over-diagnosis of autism in persons with sensory impairments [42-44]. Communicative impairment in the case of individuals with dual sensory loss is similar to symptoms of autism or mental retardation [45]. Such symptoms among children with sensory disabilities sometimes disappear, when visual, tactile or oral communication has adequately been developed. This was the case for one of the children in this study.

### Assessment and support

Like any other organic or somatic cause to mental and behavioral disorders (for example head trauma, tumour, drug abuse) assessing and treating the vision and hearing impairment is the first step in the case of Usher syndrome. Treatment of mental and behavioral disorders among children with Usher syndrome starts with developing the child's communication to reduce the negative impact of dual sensory loss [29].

Assessing mental and behavioral disorders among children with Usher syndrome using traditional tests and diagnostic procedures is often difficult due to the children's dual sensory loss and communication deficiencies [6,46,47]. Monitoring of the child's development is needed from early childhood and throughout adolescence including social, cognitive and communicative functioning in addition to vision, hearing, and balance problems. In Denmark 3-4 deafblind consultants work full time supporting children with Usher syndrome, their families, and professionals at the schools where the children attend. The service is financed by the Danish government.

### Limitations

As estimated this study only included 36% to 65% of the total population of children with Usher syndrome in Denmark, this may result in sampling error. Children with Usher type II and III, with both good vision and hearing, in mainstream schools may not be diagnosed with Usher syndrome before they reach late adolescence and are therefore not known by the national service system for children with Usher syndrome and are not included in this study. These children are probably without any serious psychological disturbances. Similarly, some children with Usher syndrome and severe mental retardation may not be assessed for vision and hearing impairment and therefore also not diagnosed or known by the national service system and thereby not included in this study. Given this possible sample error the population of this study may not be comparable with other populations.

This is a first study of mental and behavior disorders among children and adolescence with Usher syndrome. More research is needed in other populations, using other assessment tools and a control group.

## Conclusion

Different kinds of mental and behavioral disorders were found among children with Usher syndrome. Some different mechanisms that can influence the development of mental and behavioral disorders can be stated: Genes associated both with Usher syndrome and mental disorders, reaction to stress due to progressive loss of vision, and the dynamic interplay of hearing, vision, and language impairment. Monitoring the child's social, cognitive and communicative development is important for reducing the risk of developing mental and behavioral disorders. Clinical support to individuals with Ushers syndrome may be needed in childhood before the manifestation of vision impairment.

## Abbreviations

ADOS-G: Autism Diagnostic Observation Schedule-Generic; ERG: Electroretinography; RP: Retinitis pigmentosa; SDQ: Strengths and Difficulties Questionnaire.

## Competing interests

The authors declare that they have no competing interests.

## Authors' contributions

JD carried out the survey study, all data analysis and drafted the manuscript. The author read and approved the final manuscript.
